# Whole-Genome Sequence of SARS-CoV-2 Isolate Siena-1/2020

**DOI:** 10.1128/MRA.00944-20

**Published:** 2020-09-24

**Authors:** Maria Grazia Cusi, David Pinzauti, Claudia Gandolfo, Gabriele Anichini, Gianni Pozzi, Francesco Santoro

**Affiliations:** a Department of Medical Biotechnologies, University of Siena, Siena, Italy; b UOC Microbiologia e Virologia, Azienda Ospedaliera Universitaria Senese, Siena, Italy; DOE Joint Genome Institute

## Abstract

The complete genome sequence of severe acute respiratory syndrome coronavirus 2 (SARS-CoV-2) isolate Siena-1/2020 was obtained by Nanopore sequencing, combining the direct RNA sequencing and amplicon sequencing approaches. The isolate belongs to the B1.1 lineage, which is prevalent in Europe, and contains a mutation in the spike protein coding sequence leading to the D614G amino acid change.

## ANNOUNCEMENT

Here, we report the complete genome sequence of severe acute respiratory syndrome coronavirus 2 (SARS-CoV-2) isolate Siena-1/2020, belonging to the genus *Betacoronavirus* in the family *Coronaviridae*. The virus was isolated at the University Hospital of Siena (Tuscany, Italy) in April 2020, from a nasopharyngeal swab collected on 1 March 2020, and seeded on Vero E6 cells. This research was carried out according to the principles of the Helsinki Declaration, with reference to the document BIOBANK-MIU-2010, approved by the Siena University Hospital Ethics Committee with amendment no. 1, on 17 February 2020, regarding general data protection and regulation (GDPR).

After 3 days, cytopathic effect appeared on the cells, and the culture medium was collected and frozen at −80°C. Since this was the first SARS-CoV-2 viral isolate in our region, we decided to sequence it. Total RNA was isolated using the NucliSens easyMAG system (bioMérieux, Italy). Viral RNA was sequenced using both the direct RNA and amplicon sequencing approaches on a MinION instrument (Oxford Nanopore Technologies [ONT], UK).

Direct RNA sequencing was performed using the SQK-RNA002 kit (ONT). Briefly, about 300 ng of total RNA was ligated to the reverse transcriptase (RT) adapter, and the first strand was retrotranscribed using SuperScript III reverse transcriptase (Thermo Fisher); sequencing adapters were then ligated to the cDNA-RNA hybrid, and the library was loaded onto a flow cell (R9.4.1).

Amplicon sequencing was performed based on a modification of the Artic Network protocol (https://www.protocols.io/view/ncov-2019-sequencing-protocol-v2-bdp7i5rn); primers were designed using Primal Scheme (1) to generate 39 amplicons of about 900 bp with an overlap of about 50 bp (Table 1). About 100 ng of total RNA was reverse-transcribed using the SuperScript VILO reverse transcriptase kit (Thermo Fisher) following the manufacturer’s instructions and then amplified in two multiplex PCRs using PrimeSTAR GXL polymerase (TaKaRa). The samples were barcoded, pooled, and adapter ligated following the ONT ligation-based sequencing protocol. The sequencing run was managed by MinKNOW v19.12.5, enabling live base calling. For data analysis, all tools were run with default parameters unless otherwise specified. Sequencing reads were demultiplexed using Guppy v3.6.1 and then filtered using the guppyplex command of the ARTIC environment to include only reads between 700 and 1,500 bases long (https://github.com/artic-network/artic-ncov2019). Amplicon reads were mapped to the reference genome Wuhan Hu-1 (GenBank accession no. MN908947) with minimap2 v2.17 (2) and indexed using SAMtools v1.9 (3). Primer sequences were trimmed from the aligned reads using BAMClipper v1.1.1 (4). Clipped reads were then merged with direct RNA sequencing reads with the –cat command of the Linux environment. Finally, Medaka v0.12.1 (https://github.com/nanoporetech/medaka) was used to build the consensus and call the single nucleotide variants. The reference genome Wuhan Hu-1 was edited using a Perl script, selecting variants with a quality score cutoff of 35 (https://github.com/CDCgov/SARS-CoV-2_Sequencing/tree/master/protocols/CDC-Comprehensive); nucleotide variations were also visually inspected using Tablet (5). We could not sequence the nucleotides corresponding to positions 1 and 2 of the Wuhan Hu-1 genome; therefore, we obtained a 29,901-bp viral genome with an average GC content of 37.97% and a mean depth of coverage of 1,153.64×, as calculated by the SAMtools –coverage function (3).

**TABLE 1:** Primers used for amplification of the SARS-CoV-2 genome

Primer	Nucleotide sequence	Pool	Length (no. of nucleotides)	Start^*a*^	End^*a*^
CoV-2_1_L	ACCAACCAACTTTCGATCTCTTGT	1	24	30	54
CoV-2_1_R	ATGCACTCAAGAGGGTAGCCAT	1	22	867	845
CoV-2_2_L	AGTGGTGTTACCCGTGAACTCA	2	22	763	785
CoV-2_2_R	ACCTTCGGAACCTTCTCCAACA	2	22	1600	1578
CoV-2_3_L_v2	GGCTGTGTGTTCTCTTATGTTGGT	1	24	1487	1510
CoV-2_3_R	ACAATCCCTTTGAGTGCGTGAC	1	22	2414	2392
CoV-2_4_L	TTTGGCTTTGTGTGCTGACTCT	2	22	2319	2341
CoV-2_4_R	AGCAGAAGTGGCACCAAATTCC	2	22	3166	3144
CoV-2_5_L	GATTGTGAAGAAGAAGAGTTTGAGCC	1	26	3067	3093
CoV-2_5_R	CAGCGATCTTTTGTTCAACTTGCT	1	24	3878	3854
CoV-2_6_L	TCGCACAAATGTCTACTTAGCTGT	2	24	3771	3795
CoV-2_6_R	ACCGAGCAGCTTCTTCCAAATT	2	22	4658	4636
CoV-2_7_L	ACAACTGTAGCGTCACTTATCAACA	1	25	4549	4574
CoV-2_7_R	AGCATCTTGTAGAGCAGGTGGA	1	22	5359	5337
CoV-2_8_L	ACTTCTATTAAATGGGCAGATAACAACTG	2	29	5257	5286
CoV-2_8_R	AGCCACCACATCACCATTTAAGT	2	23	6172	6149
CoV-2_9_L	CCATATCCAAACGCAAGCTTCG	1	22	6019	6041
CoV-2_9_R	GCCTCTAGACAAAATTTACCGACACT	1	26	6903	6877
CoV-2_10_L	AAACCGTGTTTGTACTAATTATATGCCTT	2	29	6747	6776
CoV-2_10_R	ACTGTAGTGACAAGTCTCTCGCA	2	23	7694	7671
CoV-2_11_L	GCTTTTGCAAACTACACAATTGGAAT	1	26	7592	7618
CoV-2_11_R	GCAGCACTACGTATTTGTTTTCGT	1	24	8463	8439
CoV-2_12_L	GCGCAGGTAGCAAAAAGTCACA	2	22	8365	8387
CoV-2_12_R	TGATCTTTCACAAGTGCCGTGC	2	22	9241	9219
CoV-2_13_L	TGCTCATGGATGGCTCTATTATTCAA	1	26	9128	9154
CoV-2_13_R	GAGCCTTTGCGAGATGACAACA	1	22	9977	9955
CoV-2_14_L	GTGATGTGCTATTACCTCTTACGCA	2	25	9848	9873
CoV-2_14_R	CAGCAGCGTACAACCAAGCTAA	2	22	10688	10666
CoV-2_15_L	CAACTGGAGTTCATGCTGGCAC	1	22	10556	10578
CoV-2_15_R	GTCCACACTCTCCTAGCACCAT	1	22	11394	11372
CoV-2_16_L	TGTCTGGTTTTAAGCTAAAAGACTGTGT	2	28	11285	11313
CoV-2_16_R	ATCACCATTAGCAACAGCCTGC	2	22	12181	12159
CoV-2_17_L	GGCAACCTTACAAGCTATAGCCT	1	23	12078	12101
CoV-2_17_R	CCTACAAGGTGGTTCCAGTTCTG	1	23	12907	12884
CoV-2_18_L	GGAGGTAGGTTTGTACTTGCACTG	2	24	12793	12817
CoV-2_18_R	CGTCCTTTTCTTGGAAGCGACA	2	22	13621	13599
CoV-2_19_L	ACAGGCACTAGTACTGATGTCGT	1	23	13509	13532
CoV-2_19_R	TGGGTGGTATGTCTGATCCCAA	1	22	14328	14306
CoV-2_20_L	CAAAGCCTTACATTAAGTGGGATTTGT	2	27	14224	14251
CoV-2_20_R	GGTGCGAGCTCTATTCTTTGCA	2	22	15108	15086
CoV-2_21_L	AGGATCAAGATGCACTTTTCGCA	1	23	15004	15027
CoV-2_21_R	AGTAAGGTCAGTCTCAGTCCAACA	1	24	15858	15834
CoV-2_22_L	TGCATCTCAAGGTCTAGTGGCT	2	22	15749	15771
CoV-2_22_R	GCGTTTCTGCTGCAAAAAGCTT	2	22	16648	16626
CoV-2_23_L	CGATAATGTTACTGACTTTAATGCAATTGC	1	30	16535	16565
CoV-2_23_R	GTGCAGGTAATTGAGCAGGGTC	1	22	17458	17436
CoV-2_24_L	TCTTTGATGAAATTTCAATGGCCACA	2	26	17350	17376
CoV-2_24_R	GCTTCTTCGCGGGTGATAAACA	2	22	18275	18253
CoV-2_25_L	TGGCATACCTAAGGACATGACCT	1	23	18167	18190
CoV-2_25_R	ACCAATGTCGTGAAGAACTGGG	1	22	19038	19016
CoV-2_26_L	TGATGAACTGAAGATTAATGCGGCT	2	25	18938	18963
CoV-2_26_R	GCAGCAATGTCCACACCCAAAT	2	22	19862	19840
CoV-2_27_L	ACACAAAAGTTGATGGTGTTGATGT	1	25	19714	19739
CoV-2_27_R	GGTTGCCACGCTTGACTAGATT	1	22	20678	20656
CoV-2_28_L	TCTGTAGTTTCTAAGGTTGTCAAAGTGA	2	28	20553	20581
CoV-2_28_R	AAAGACATAACAGCAGTACCCCTTAA	2	26	21443	21417
CoV-2_29_L_v2	CAAACCACGCGAACAAATAG	1	20	21297	21316
CoV-2_29_R_v2	CGAAAAACCCTGAGGGAGAT	1	20	22225	22206
CoV-2_30_L	AAACAGGGTAATTTCAAAAATCTTAGGGAA	2	30	22105	22135
CoV-2_30_R	TGTGCTACCGGCCTGATAGATT	2	22	22996	22974
CoV-2_31_L	AACAATCTTGATTCTAAGGTTGGTGGT	1	27	22876	22903
CoV-2_31_R	TGCTGCATTCAGTTGAATCACCA	1	23	23813	23790
CoV-2_32_L	ACCCACAAATTTTACTATTAGTGTTACCAC	2	30	23703	23733
CoV-2_32_R	TGCACTTCAGCCTCAACTTTGT	2	22	24537	24515
CoV-2_33_L	TGCACAAGCTTTAAACACGCTT	1	22	24426	24448
CoV-2_33_R	GCAGCAGGATCCACAAGAACAA	1	22	25324	25302
CoV-2_34_L	CTAGGTTTTATAGCTGGCTTGATTGC	2	26	25213	25239
CoV-2_34_R	ACATGTTCAACACCAGTGTCTGT	2	23	26075	26052
CoV-2_35_L	GGGAATCTGGAGTAAAAGACTGTGT	1	25	25969	25994
CoV-2_35_R	AATGACCACATGGAACGCGTAC	1	22	26857	26835
CoV-2_36_L	TGGATCACCGGTGGAATTGCTA	2	22	26744	26766
CoV-2_36_R	GTGTTTTACGCCGTCAGGACAA	2	22	27612	27590
CoV-2_37_L	ACGAGGGCAATTCACCATTTCA	1	22	27511	27533
CoV-2_37_R	ACTGCCAGTTGAATCTGAGGGT	1	22	28351	28329
CoV-2_38_L	AGAGTATCATGACGTTCGTGTTGT	2	24	28219	28243
CoV-2_38_R	GCTTCTTAGAAGCCTCAGCAGC	2	22	29045	29023
CoV-2_39_L	TGCTTGACAGATTGAACCAGCT	1	22	28940	28962
CoV-2_39_R	TTCTCCTAAGAAGCTATTAAAATCACATGG	1	30	29866	29836

a Nucleotide positions relative to the Wuhan Hu-1 reference genome.

**FIG 1:**
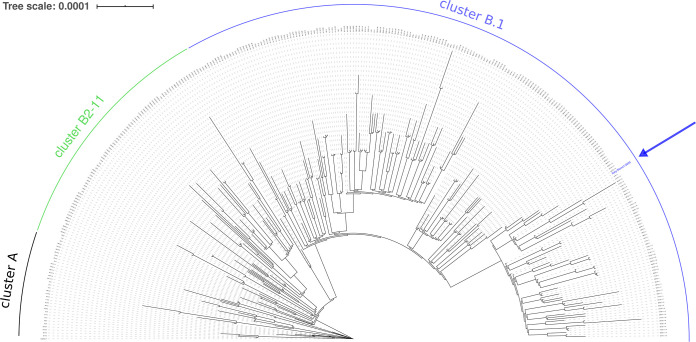
Phylogenetic tree of SARS-CoV-2 genomes. The tree was generated with Pangolin v1.14 and visualized using Interactive Tree Of Life (iTOL) (8). A total of 322 viral genomes are displayed, including the genomes selected by Pangolin software as representatives for the genetic diversity of SARS-CoV-2. As of 30 July 2020, two major clusters (A and B) were identified. Cluster B was subdivided into 11 clusters (B1 to B11); of those, the most represented is cluster B1 (covered by the blue arch), which comprises most of the lineages identified and sequenced in Europe. Cluster B1.1 is a large subcluster characterized by the mutation of three consecutive nucleotides at position 28881. The blue arrow indicates the position of the Siena-1/2020 isolate in the phylogenetic tree.

Phylogenetic analysis performed with Pangolin v1.14 (6) assigned strain Siena-1/2020 to the B1.1 lineage, which is associated with the Italian SARS-CoV-2 outbreak and includes isolates circulating in Europe (Fig. 1). Compared to the reference genome Wuhan Hu-1, Siena-1/2020 harbors 5 single nucleotide changes (at positions 241, 3037, 14408, 19839, and 23403) and mutations of 3 consecutive nucleotides (GGG→AAC) at position 28881. Among the 5 single nucleotide changes, the one at position 23403 causes a change in the predicted amino acid sequence of the spike (S) protein (D614G), which is now the most common variant worldwide (7).

### Data availability.

The genome sequence of SARS-CoV-2 Siena-1/2020 has been deposited in GenBank under the accession no. MT531537. The version described in this paper is the second version. The raw Nanopore reads were deposited in the Sequence Read Archive under BioProject accession no. PRJNA658490 with accession no. SRX8982904 (direct RNA sequencing) and SRX8982905 (amplicon sequencing).
